# The Violence of the Cut: Gendering Self-Harm

**DOI:** 10.3390/ijerph18094650

**Published:** 2021-04-27

**Authors:** Amy Chandler, Zoi Simopoulou

**Affiliations:** School of Health in Social Science, University of Edinburgh, Edinburgh EH8 8AG, UK; zoisimopoulou@yahoo.co.uk

**Keywords:** self-harm, gender, art

## Abstract

Taking as a starting point the frequent characterisation of self-harm as “an adolescent thing for girls,” this paper offers a sociologically informed, qualitative exploration of self-harm as a gendered practice. We move beyond statistical constructions of this “reality,” and critically examine how this characterisation comes to be, and some of its effects. Our data are drawn from a pilot study that developed a collaborative arts-based inquiry into meanings of self-harm. The authors worked with two groups: one of practitioners and another of people who had self-harmed, meeting over six sessions to discuss and make art in response to a range of themes relating to the interpretation and explanation of self-harm. Through data generation and analysis, we collaboratively seek to make sense of the gendering of self-harm, focusing on a series of dualistic Cartesian “cuts” between male and female, violence and vulnerability, and inside and outside. In conclusion, we call for more multi- and interdisciplinary explorations of self-harm, and greater use of diverse, arts-based, and qualitative methodologies, in order to further expand and nuance understandings and ethical engagements with self-harm, and those who are affected by it.

## 1. Introduction

“I do wonder why that’s happened, why it has been viewed as an adolescent thing for girls” (Anna, practitioner, 2018).

As Anna, a participant in our study, notes, self-harm is often seen as “an adolescent thing for girls.” This understanding ties the practice to a particular gender identity, a certain age. Added to this—and often unspoken—is that self-harm is framed as a practice of adolescent girls who are also white [1]. Each of these claims is subject to challenge by existing studies. Depending on the definition of self-harm used and the population studied, between one-fifth [2] and one-third of those reporting self-harm identify as male [3], with some studies finding near-equal rates [4]. Concluding that “being female is a risk factor for self-harm” obscures other readings, other realities. In terms of age, rates do seem higher among younger groups, and some studies suggest that most people who self-harm as teenagers stop by the time they are in their 30s [5]. However, studies also show that self-harm can begin in later life, or continue across the life course, and may be significantly under-reported among older groups [6,7]. Where studies engage with race (and often they do not), findings are varied—some studies in UK contexts find no evidence of patterns by ethnic group [3]; however, one study based in the Southern US found that the highest reported rates were among black males [8]. Patterns of self-harm are diverse, sensitive to context, definitions, and measures used. Despite this, in the public imaginary, and in media representations [9], self-harm is very frequently cast—as Anna notes—as “an adolescent thing for (white) girls.” We wonder why as well. 

Studies that attempt to map the epidemiology of self-harm have historically used a range of definitions—self-mutilation, self-injury, nonsuicidal self-injury, deliberate self-harm, and more [10,11,12]. The practices these definitions refer to also vary. In much UK-based research, the term “self-harm” refers to “self-injury or self-poisoning, irrespective of the apparent purpose of the act” [13]. However, depending on the research setting, this broad definition can lead to some confusion [10]. In hospital-treated self-harm, around 75% of patients have taken overdoses [14]. In contrast, in community studies (often educational settings), using self-report measures, around 60% of young people who report self-harm describe self-cutting [15,16]. Each of these practices may have different meanings. Further confusion can arise when determining whether an act of self-harm is an act of “attempted suicide” or not. Determining intent is avoided in much UK-based research, yet “deliberate self-harm” is often conflated with “attempted suicide.” Qualitative research indicates that meanings and motivations of self-harm can vary and change over time [17]. Many studies do not report on methods of self-harm [3], raising questions about claims made. This has implications for understanding a relationship between gender and self-harm, since the nature of this relationship varies according to the type of study and its context (e.g., hospital or community sample), how self-harm is defined (broadly or more specifically), and the age group/s included. 

Recently, concerns have been raised about an apparently stark rise in the numbers of young women reporting and being treated for self-harm [3], rising numbers of men and women being treated for self-cutting in hospital settings [14], and signs that rates of suicide are rising particularly among young women (aged 16–25) [18]. While we might want to question the definitions used and populations studied, it does seem clear that *something* is happening. However, it is also clear that whatever is happening in terms of gender and self-harm builds on a long history of self-harm being framed and understood as a female act. Therefore, we must understand these latest developments in this light.

Historical work has demonstrated how meanings of self-harm have varied over time [1,11,12]. These studies highlight the influential work of a particular group of psychiatrists working in the US in the 1960s. Brickman and Chaney show how this group of practitioners and researchers worked hard to construct—rather artfully—self-injury (or “delicate self-cutting,” as they called it) as a *feminine* practice. Published papers routinely excluded men from their analysis as “not typical patients,” or described them as “effeminate.” Brickman argues that the reason these researchers worked so hard (against evidence in some cases) to frame self-injury as a feminine practice was that the alternative might be to accept that women could engage in “aggressive” or “violent” (“masculine”) acts. This analysis questions the naming of the practice as “delicate”—unsettling the use made of language that seeks to artfully feminise a practice that might just as easily be read as violent, aggressive, and masculine. 

Aside from these critical reviews of historical clinical literature, and despite the frequently gendered findings regarding self-harm, attempts to more deeply explore the relationship between gender and self-harm have been surprisingly scarce [19,20]. This situation is exacerbated by studies that frequently focus only or mainly on accounts of women, with self-harm read as a largely female practice, without much further interrogation [10]. Where a more explicit attempt is made to examine gender in relation to self-harm, this has often resulted in a focus on women’s oppression and experiences of abuse and violence [20]. However, as Brossard [21] has noted, a focus on sexual violence as an explanation for self-harm can become a “total explanatory discourse” (p. 151), where abuse/violence provides *the* answer, and no other interpretations are sought out. This is problematic, because it takes for granted and does not seek to further interrogate *why* there might be a relationship between experiences of abuse and the enactment of violence or harm against the self. Further, it leaves unanswered how and why people may come to self-harm in the absence of such experiences. 

Another important way in which self-harm is gendered is via the gendering of suicide [22]. Canetto and Sakinofsky [23] popularised the concept of the “gender paradox” of suicide—whereby men are seen to predominate in statistics on suicide, while women predominate in statistics on self-harm. This early work underlined the cultural basis of the paradox, with men’s and women’s differing engagements in suicidal practices (with different outcomes in terms of lethality) tied to gendered cultural expectations of how men and women should be. Crucially, they argued, women’s self-harm has often been framed as “attempted” or “failed” suicide. Such an interpretation builds on cultural ideas of masculinity (as active, aggressive, successful) and femininity (as passive, weak, less than). As we discuss below, while many of these assumptions have been strongly challenged, elements of these can still be identified in how self-harm (and suicide) is discussed in contemporary literature and public debate [24,25]. In particular, this can be seen in the endurance of the notion of “attention-seeking” self-harm, and this being associated with “hysterical” young women [26,27]. 

In this paper, we interrogate the construction of meanings about self-harm as gendered, and we consider the effects of such constructions as illustrated and enacted by ourselves and our participants within collaborative arts-based workshops. In doing so, we seek to show the role of socially mediated, cultural meanings of self-harm in producing it as gendered. This approach goes beyond statistical approaches that construct self-harm as gendered via findings that show that “self-harm is more common in girls”—and looks to other ways in which self-harm is gendered, made sense mainly as a practice of young (white) women. We argue that attending to meaning as well as prevalence is essential if we are to develop responses to self-harm that trouble rather than reinforce oppression and control. 

## 2. Materials and Methods

This was a pilot study testing a novel method. We sought to explore meanings of self-harm through a combination of group discussions and art making. The project was designed in response to concerns that surveys or qualitative interviews tend to fix meaning, resulting in increasingly standardised explanations for self-harm that do not well reflect the complexity of experience and meaning that self-harm can involve [17]. Our project was informed by calls within sociology for greater use of “live” methods [28,29], and by the burgeoning field of arts-based research [30]. These are methodological approaches that are “lively,” making space for creativity, innovation, and ongoing relationships among participants and researchers. Such approaches recognise that social life and meaning may not be easily “captured” by interviews or surveys. Further, they allow explicit attention to how meanings are produced through social interaction. Indeed, arts-based approaches enable engagement with the shifting and often unfixed nature of meanings—how we feel or talk about a given topic may change over time, according to context, to the words and means available to us to communicate. Rather than attempt to access a (falsely) “neutral” or “objective” view of a participant’s life-world or understanding of a topic, live and arts-based approaches embrace complexity, contradiction, and nuance as the focus of enquiry [30]. 

In the study, we were concerned with exploring meanings of self-harm among people who had a stake in the issue: people who had self-harmed and practitioners working with those who have self-harmed. Acknowledging the limitations of words in articulating meaning, we designed the study to make space for nonverbal means of meaning making—informed by Tarr and colleagues’ arts-based research workshops on pain [29] and Foster’s collaborative arts-based research [31]. In our study, we used visual materials—paint, pencils, pens, and paper. 

We held six art- and discussion-based workshops with two separate groups in a UK city. The first group included two practitioners, both white cis women who worked for a mental health organisation and supported people who had self-harmed through one-to-one therapeutic work. The second group included three white young people—two cis women and one nonbinary person—recruited through the same mental health organisation, who were aged 21–26 and who had all previously self-harmed. Two of these participants indicated they had last self-harmed over a year previously, with one participant indicating this was an ongoing practice. With each group, our workshops were based around the same themes (Box 1).

Box 1 **Workshop themes.**

1. Communication, silence, and privacy
2. Skin and pain
3. Ritual
4. Gender and culture
5. Life course, age, and culture
6. Open session

For each workshop, researchers brought a selection of four to five materials, mostly excerpts from publications, but occasionally images and videos, that presented different perspectives on a particular theme (Box 1). The materials were selected carefully, drawing on a previous review of literature on the theme of “creativity and self-harm” [32]. Our aim was to encourage participants to reflect critically on different perspectives, explore their thoughts and feelings about them in the light of their own experiences, and “respond” to them by means of both art and words. As such, we sought to open up space for a nuanced discussion around a topic, rather than seeking to fix one meaning or interpretation of self-harm as the “right one.” 

Workshops lasted 90 min, beginning with a 30 min recorded discussion about that session’s theme and materials. These discussions began with each of us taking turns to read one of the short excerpts. This was initially offered as a suggestion (not a requirement), designed to allow each person to speak at the very start of a workshop and bring their voice into the room. In practice, all participants were happy to take part in this, though we imagine that not all groups would have similar levels of literacy, confidence, or comfort to allow for this type of engagement. After 30 min of initial discussion, the recorder was switched off, and we spent 30 min of quiet art making. A selection of materials was made available, and each of us worked individually to explore the theme further through art making. No expectations were placed on artistic ability, and a supportive atmosphere was generated by both Zoi’s expertise as an art therapist and Amy’s explicit presentation as someone who did not “do art.” That said, all participants were clearly comfortable and experienced with art making, which likely guided their initial interest in taking part in the project. After 30 min, we switched the recorder back on for a final 30 min discussion, during which we shared what we had made and reflected further on the topic of the week. 

Our data comprised approximately 1 h of recorded talk per workshop, which was transcribed (12 h in total); fieldnotes from both authors; and the pieces of art made by the researchers and participants. Analysis of these diverse forms of data has drawn on the principles of feminist poststructuralist qualitative inquiry [33] and writing as a method of inquiry [34]. For the present paper, Amy read and reread (or viewed) all data from the project, drawing out aspects of the data that spoke implicitly or explicitly to the theme of gender. Particular attention was paid to Workshop 4, which had focused on gender. 

The dynamics of the group and our analysis of the data are invariably shaped by our own positionality and experience in relation to self-harm. Amy is a sociologist who has previously self-harmed and has researched the topic professionally for many years, while Zoi is an academic in counselling and psychotherapy, as well as a practicing art therapist who has worked with young people who have self-harmed but came to the topic as an academic more recently. Thus, both researchers brought different types of knowledge of self-harm to the research. 

The study was assessed by the Institutional Research Ethics Committee. All the participants chose pseudonyms and provided informed written consent to take part. We approached ethics as an ongoing, negotiated, and relational process. This included checking in on participants’ well-being before and after workshops and maintaining contact between and after the workshops were completed. We met with both groups prior to publicising any findings, and all the participants joined us at public engagement events, where we shared and discussed initial findings. Thus, while we focus on our engagement with the participants around the workshops in this paper, we have benefitted from ongoing relationships with the participants as part of a relational ethical praxis [31,33]. 

In the remainder of this paper, we introduce three aspects of the gendering of self-harm, which we drew out of the data, via writing as inquiry and through our collaborative work within the groups. In presenting the findings, we make clear the intertwined roles of art, discussion, and our own analytic and imaginative reflections [35] in producing, interrogating, and unsettling knowledge about the gender of self-harm. 

## 3. Results

Gender was a frequent presence—and absence—within our discussions. Even during the workshop where gender was the main topic, a notable feature was the difficulty that all of us—participants and researchers—had in talking directly to gender. Thus, one of the themes we draw out is “unsettling talk about gender and self-harm,” which we illustrate through an exchange with Jessica, one of the practitioners, who made this difficulty visible in her art. Through this, we introduce the presence of several binary oppositions that characterised our attempts to make sense of self-harm as gendered. These included aggression and vulnerability, inside and outside, and through these, the binary oppositions that are invoked when considering self-harm as “masculine” or “feminine.” Throughout this analysis, we are mindful of Whynacht’s [36] cautions about the “violence of the cut” that such binaries generate, the splitting off of one thing (nature/culture; self/other) from another in a way that silences complexity and nuance.

### 3.1. Bringing and Drawing Themes

Before turning to these more substantive issues, we introduce the resources we brought and the images that we created with the participants during Workshop 4, where we explicitly addressed the theme of gender. These are instructive and help to show how this theme took shape within our discussions and later writings into what happened in these workshops. 

Our materials for the workshop on gender included excerpts from Brickman [1], Inckle [37,38], and Shaw [39]. Brickman and Shaw each presented critical, feminist-informed analyses of the focus on women found in much clinical literature on self-harm, and Inckle’s [37,38] work drew on interview studies with women and men. We also brought a poem titled “Hurting Myself” [40], published in *The Cutting Edge: A Newsletter for Women Living with Self-Inflicted Violence.* The poem was confronting and explored themes relating to gender, gendered oppression, racist and sexist violence, as well as challenging interpretations of “self-harm” as being about hurting oneself. 

**Figure 1:**
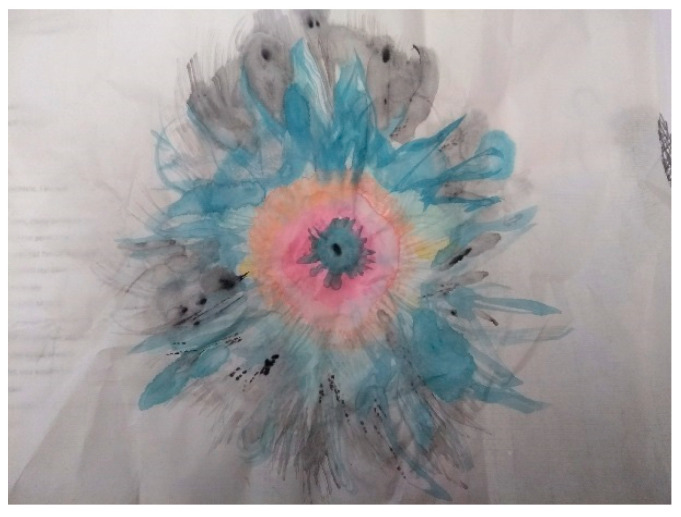
Jessica, Session 4.

In our first group, Anna and Jessica, the two practitioners, created images that included an abstract, flowerlike, circular, messy image (Jessica, see Figure 1); a picture of a stick woman with her back to a stream of red crayon splitting the page (Anna, see Figure 4); a collage of words cut out and repurposed from the readings (Amy); wraithlike pencil drawings of people, some carrying burdens on their backs, which overlaid the words of the readings (Zoi).

**Figure 2:**
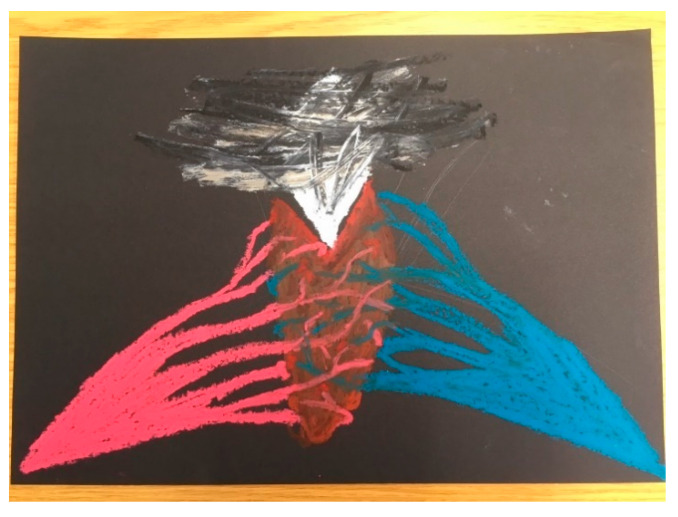
Jo, Session 4.

**Figure 3:**
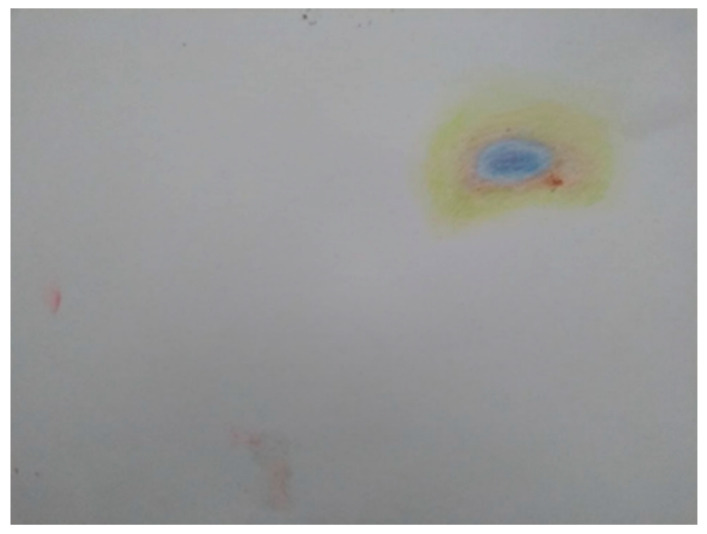
Amber, Session 4.

In our second group, with Chloe, Jo, and Amber, the images that were created included a brick wall with hands and a screaming face pushing through a gap (Chloe); an abstract image of two handlike or veinlike objects coming together, coloured pink and blue, with the centre a thickly rendered grey explosion (Jo, see Figure 2); a small and carefully drawn “bruise” on an otherwise blank piece of paper (Amber, see Figure 3); an abstract oval shape built up with multiple layers of paint (Zoi); another collage of words, this time made into a poem of sorts (Amy). 

### 3.2. Unsettling Talk about Gender and Self-Harm

In both the quote and the image (Figure 1), Jessica attempts to articulate something of the difficulty that was palpable across both groups in “talking” about the relationship between gender and self-harm.

*“As I was doing the artwork, I had the same problem as you, I found it very hard to talk about, find the words for, or to think about in a way, and kind of ignored in some ways the gender, things like that were too difficult somehow, but felt like there was somewhere within that conflict, there seems to be tension between vulnerability and sort of aggression, and a relationship between the two, ’cause I think they’re joint, and I was interested in how, sort of people perceive that and just that there is back and forth between the two, not necessarily linked to gender but linked to maybe how people see gender and not, yeah, part of it being quite, unsure what is what.”*

Jessica

In our fieldnotes, both authors noted the “heaviness” this topic brought to the room. Our workshops were often marked by periods of silence and reflection, but both of us noted a different, harder quality when we attempted to discuss gender. This difficulty speaks to a challenge that the discipline of sociology grapples with regularly: how to connect individual experiences (private troubles) with larger social structures (public issues) [41]. 

This was emphasised in the second group, where we discussed how limiting a view of self-harm as “female” or “male” was—Chloe underlined self-harm as personal, which made it difficult to apply a binary of “male or female”:

*“At the same time self-harm is very personal, is like not a gendered thing, I mean like it may depend on so many things in your character or your personality or you know that you could probably find a group of men, or women doing that in the same way and then yeah I don’t know it doesn’t seem, those claims, assuming that ‘this is that,’ ‘this is that,’ it just sounds so limited, so small, so, yeah even as you’re saying, you’ve got to choose, this or that, there’s nothing in between, is like no space for, for confusion.”*

Chloe

Indeed, the inherent limitation of developing explanations or making sense of self-harm using a binary definition of gender was further challenged by Jo as they discussed their image (Figure 2):

*“I’ve got the masculine and man and feminine and womanhood like being seen as opposing forces, being seen as opposite and different and the only options, and I think I guess I felt a little bit, invisible within the readings, and having dealt with that, kind of, feeling pulled and having to choose and having to align myself ’cause even in like being nonbinary, it sounds like are you transmasculine or are you trans-feminine, and it’s like no, I am nothing, I am—no, I am not that, I am not within that kind of context, and it’s painful to kind of have those opposing kind of forces and then the idea of being like ripped open and revealing your kind of inner self, […] and I accidentally bent the grey pastel in half (laughs) so I apologise, I got a bit violent at the end…”*

Jo

Jo’s image (Figure 2) and discussion following spoke to their frustration and pain in trying to consider both themself and their self-harm in the context of binary gender. Read alongside Whynacht’s [36] examination of the “Cartesian cuts” between mind and body, self and other (p. 5), Jo’s image and words point to another violent “cut” between male and female—one that separates and sets an opposition between two ways of being, neither of which seem sufficient. 

### 3.3. Violence and Aggression 

Violence and aggression are key resources through which self-harm is gendered in existing research and commentary. Brickman [1] and Chaney [11] both identified how in early clinical work, self-cutting was framed as a “delicate” practice, rather than one of violence and aggression, which were seen as masculine and therefore incompatible with a practice framed as “feminine.” Across both groups, we toyed with ideas around self-harm, aggression, and violence, including whether methods of self-harm might relate to gender. 

For instance, each group spoke to the idea that men may be more likely to use fighting as a form of or alternative to self-harm. This suggestion serves to blur the very notion of *self*-harm, with fighting or violence against others necessarily involving *other*-harm as well as self-harm.

*Jessica: I don’t know if this is based on anything whatsoever (laughs), but often people do say […] you know […] is a female girl thing, but it’s this thing that I have heard and probably said it myself too […] that typically women turn things inwardly while men take things—*

*Anna: Fight*

*Jessica: Fight, externalise them more, and I don’t know that’s just sort of something like that they say I have no idea if there is any basis for that.*

*Anna: It’s easier to hide self-harm if you’re a rugby player and you come out all bruised and battered, there’s something like you can’t hide that, whereas maybe women don’t have the same exposure to the physical sports that they could get that release from… …I don’t know, maybe that’s how men have found certain ways of self-harming and women have found other ways of self-harming.*

This argument was present in the reading we shared by Inckle [37]. One of Inckle’s participants spoke about how his self-inflicted injuries had been assumed to be caused by injuries from fighting, with her analysis suggesting that such assumptions contributed to the invisibility of male self-injury, especially among young working-class boys. 

How wounds or injuries are interpreted by others makes up another route through which self-harm—and suicide [22]—is gendered: whether injuries are interpreted as “delicate” or “coarse” [11], who is assumed to have caused the injury, what meanings or motivations are imputed. All of these are infused with gender. Though she expressed some hesitation about how relevant her picture was to the theme of gender, we think these issues are illustrated well by Amber’s picture of a bruise (Figure 3). 

Amber’s picture of the bruise (Figure 3) holds something ambiguous: it is sharp both in how carefully it is made and in the pain that it conveys, yet the different shades fade into each other, giving a sense of something almost powdery. The bruise is drawn on an otherwise blank page.

*“I ended up thinking about, more of the how different types of self-harm are viewed as more acceptable, thinking about the bruising aspect, I coloured in a bruise (showing her artwork), […] and yeah, I am not really sure how that fits in with identity and gender, […] I mean I don’t know if this is related to the subject at all, but, the kind of thought that bruising is very easily interpreted as being inflicted by someone else, whereas cutting is a lot more kind of obviously well maybe self-inflicted, but that sometimes kind of the reaction to that it’s, one realises that something that they thought someone else did to you was actually what you did to yourself, they can be quite shocked by that, I had a nurse once that saw some bruises on my rib cage, and she asked, ‘Someone else did it?’ And I said no, that was me, and she was quite concerned by that.”*

Amber

The bruise on the paper and Amber’s bruises in her accompanying story were subject to multiple readings. A nurse asked if “someone else did it.” This question—assumption perhaps—that someone else must have caused harm to Amber’s rib cage speaks to the more usual way in which violence is gendered when read on a female body: it is assumed to have come from “another,” often a “male other.” When enacting violence both on the self and on others, violence and aggression from women elicit shock, disbelief, disgust—pathologisation [42]. 

Writing after the workshop had ended, Zoi recalled Amber’s bruise and her story:

*“Amber speaks of how ‘something they thought that someone else did to you was actually something you did to yourself.’ She says that people can be quite shocked by that. I feel shocked. I tell her, about the violence towards the self as the violence towards the woman or towards the woman who harms herself. She stays silent. I feel anxious. I look for her eyes to ease my worry that I have just harmed her with my words.”*

This reflection draws attention to the relational way in which we make meanings out of self-harm between ourselves. These meanings are not fixed—they are alive [28]—and the meanings are also heavy with moral weight. Zoi worries about harming Amber with her words. We might draw out harm from assumptions made by nurses, which close down alternative—more difficult—explanations for how a bruise comes to be. 

Amber notes that cutting—which she had also done—was much less subject to this type of interpretation. Self-inflicted cuts were “more kind of obviously … self-inflicted.” This is important to notice; cuts are indeed more easily viewed as self-inflicted. As Amy wrote in her notes following this workshop, *“a bruise is that much more ambiguous than a cut.”* However, we wonder if in part this relates to the *type* of body—gendered, raced—on which the injuries are seen. Would Amber have been asked whether “someone else did it” if she were male, older, not white? Perhaps on other bodies, questions would be asked differently, or not at all.

### 3.4. Inside and Outside

Closely related to discussions of violence and self-harm were the similarly dualistic notions of “inside” and “outside.” This is seen in Anna and Jessica’s exchange about *a* “common-sense” idea about men and women where “women turn things inwardly” while men externalise—they “fight.” This discussion has parallels in existing literature about gendered distress and mental health, which draw on similar imaginaries [12,43,44]. Men are understood to repress emotions, not talking so often about distress, whilst simultaneously being seen as more liable—“allowed”—to express violent or aggressive emotions on other people in ways that are less socially acceptable for women [45]. 

The idea that distress is contained in the body, until it becomes too much and is “released” in “violent activity” is traced by historian Millard [12] (p. 167) to early research with “female psychopaths” in the 1960s by clinical psychologists working in closed settings. In our discussions, this way of understanding self-harm was also present. Whether self-harm might be a version or proxy of such release was reflected by Jo.

*“It made me think about the acceptability of expressing emotions outwardly and conscious of how maybe it is a misconception of that social acceptability of being able to lash out or have a fight or go and play rugby really violently and feeling quite constrained, ’cause I know for me not being able to express anger especially was a massive thing that led me to turn that in on myself, and not having an outlet, where it was kind of like you are a woman, you shouldn’t be getting angry and shouting and slamming and fighting, not that that would necessarily be a better way of doing it, but it felt, to me this translates to any anger at all is not OK, and that was not helpful, like not at all, so I wonder if that ties to, a little bit as well, that turning inwards of anger.”*

Jo

This discussion points to further paradoxes in how self-harm, emotions, and gender are made sense of. It takes us back to wondering whether self-harm is “violent” or not. Is it a way of “expressing” or “internalising” anger? A response to oppression or a reaction against oppression? Self-harm embodies contradictory notions of expression and repression—read in some cases as a way of expressing and communicating strong feelings (including anger), and in others as a secretive act of emotional *self*-regulation. In each case, this is done by making something imagined “internal” into something more “external”—on the skin or against others. Importantly, while Jo astutely highlights the role of emotional cultures here, in many cases, the source of anger is framed as the person (often female) who self-harms. 

**Figure 4:**
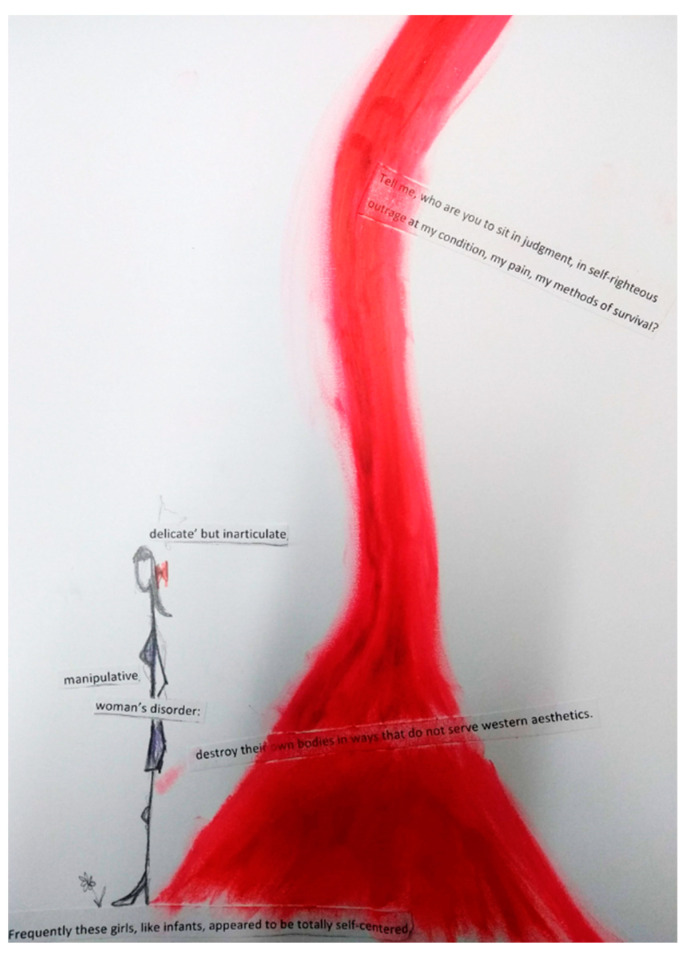
Anna, Workshop 4.

These contradictory notions of oppression or explosion can be read into both the image that Anna created (Figure 4) and our subsequent discussion. 

This image (Figure 4) was one that we returned to frequently in our analytic writing on self-injury. 

Zoi wrote after the workshop:

*“I see the strong body of red crayon, same colour as the tiny hairband on the woman’s hair. I imagine the hairband breaking open and the red wave flowing forcefully from it. There is an aliveness. ‘Let your hair down.’ Wildness. Rawness. Aggression. Femininity. Wound? Anna says that the woman stands with her back to it. She speaks often today about ‘thoughts that are not fully formed.’ I recall a feeling of sadness, something vain or already lost; we move slowly. I draw women carrying; raising: genders, burdens, children, ‘white powerful men.’ Anna reads in my drawing something ‘disconnected.’ I find it hard to find words to speak about how gender is present as if it is something nebulous. Yet, I feel the room crowded. Gender is peopled. We are here with our bodies. […] There is something fleshy that runs through the narratives as we tell ourselves in the room. Our bodies—streams of red crayon—cannot be held back from how we read or how we are read.”*

Within the workshop, the “body of red crayon” was read differently—Amy saw an explosion, a volcano, red fire—blood—streaming upwards and out. From slightly different vantage points in the room, the others all saw this initially as a red liquid pouring *down*. A more oppressive move, perhaps.

The different readings of Anna’s image recall the different interpretations of Amber’s bruise (Figure 3). Rather than close down or simplify such contradiction, we suggest that these readings can and must sit together simultaneously.

## 4. Discussion

In *The Gender of Suicide*, Jaworski [22] asks “whether analysing gender could change how we know suicide” (p. 156). In this paper, we have begun to explore whether analysing gender might change how we know self-harm, or perhaps more precisely, whether considering the ways that self-harm is constructed as gendered might change how we make sense of self-harm. In doing so, we have begun to examine not just the ways that self-harm is gendered—through frequent invocation of Cartesian cuts and binary oppositions—but also some of the effects and implications of this gendering. There is—as Whynacht has articulated—violence in these cuts [36,46]. The images made in our workshops, especially on gender, spoke to this violence and its effects—to oppression, to being trapped, and to explosions that can happen in response. 

Recent writing on self-harm has emphasised the importance of bodies, relationality, and repetition in making sense of the practice. This has included considerations of the binary oppositions that we have sought to unsettle in this paper in terms of how self-harm is gendered. Steggals and colleagues, reflecting on the importance of a more relational and sociological interpretation of self-harm, suggest that:

“The monadic, self-sufficient individual, or homo clausus (Elias, 2000 [1939]), and the whole binary structure of inner/outer, individual/social and private/public that goes with it (Callero 2009, Derrida 1982) must be reframed as patterns of enacted and embodied values rather than as ontological givens” [47] (p. 168).

Considering such binaries as “enacted” is useful and provides a way of understanding them as produced rather than “natural.” Heney [19] takes this point further in her analysis of how self-harm can contribute to feminist theorisation of agency as also embodied and relational. She highlights the role of repetition—that self-harm and negotiations of agency are not singular, one-off assessments. They are “enacted and embodied,” as per Steggals et al., but also repeated—remade and renegotiated. We note that interpretations and meanings of self-harm are also “enacted and embodied” and repeated. Together, these insights help us to notice the fleshy, relational, and ultimately tentative nature of the meanings self-harm is understood to have. In our workshops, this messy relationality was given space and privileged. We suggest that this analysis challenges, and in so doing adds to, existing quantitative and qualitative studies of self-harm, where meanings of the practice, and its relationship to gender, are more often taken for granted or at least viewed as more stable.

As a pilot study, drawing on close and collaborative work with two small groups of participants, our findings and discussion are inherently limited. In particular, our groups were composed mostly of white cisgendered women, though undoubtedly conversations were enhanced by input from Jo, who is nonbinary, and helped us to acknowledge and raise questions about our own assumptions and those present in existing literature. The richness of the data we were able to generate together and the subsequent depth and nuance to our analysis would have been harder to attain with a larger sample—this is one of the strengths of qualitative research. That said, we suggest that our approach could and should be tried with more diverse groups of people. A significant gap in existing research on self-harm, which we have now further contributed to, relates to race and ethnicity—we have maintained an uncomfortable focus on whiteness, which is largely unmarked here [48].

## 5. Conclusions

Using art and words and bringing materials that invited complexity into the room, our workshops successfully made space where participants were able to explore meanings of self-harm without “fixing” them. In doing so, we were guided by Back’s call for research that seeks to “account for the social world without assassinating the life contained within it” [28] (p. 21). Social life is messy and complex, and such approaches allow us, as researchers, to engage with this with participants—to “show” some of this complexity as we also seek to avoid fixity. 

However, complexity aside, there is much at stake when considering the gender of self-harm. The heaviness we felt in the room during our sessions discussing gender, and the pain, discomfort, and oppression that the participants recognised in the reading materials and connected to their own lives attest to this. We suggest that a further benefit of our use of arts-based, “live” methodologies to study meanings of self-harm and gender is the potential capacity for social action and transformation. On a small scale, the workshops offered a space where alternative meanings of self-harm could be repeated and practiced and where more dominant readings of gender could be challenged. Though this is a hugely fraught area, as Foster [31] has explored in detail, we see much to feel hopeful for in these methods and in their capacity to work with collaborators to reimagine and repeat alternative meanings of self-harm.

## Data Availability

Due to the sensitivity and specificity of the data generated, data from this study are not publicly available.
